# A Mutation in Endogenous saRNA miR-23a Influences Granulosa Cells Response to Oxidative Stress

**DOI:** 10.3390/antiox11061174

**Published:** 2022-06-15

**Authors:** Siqi Wang, Yuqi Li, Qiang Zeng, Liu Yang, Xing Du, Qifa Li

**Affiliations:** College of Animal Science and Technology, Nanjing Agricultural University, Nanjing 210095, China; 2019105018@njau.edu.cn (S.W.); 2021105015@stu.njau.cn (Y.L.); 2019105019@njau.edu.cn (Q.Z.); 2019205011@njau.edu.cn (L.Y.); duxing@njau.edu.cn (X.D.)

**Keywords:** miR-23a, GC apoptosis, saRNA, *NORHA-FoxO1* pathway, mutation, oxidative stress

## Abstract

Phenotypes are the result of the interaction between the gene and the environment, so the response of individuals with different genotypes to an environment is variable. Here, we reported that a mutation in miR-23a influences granulosa cells (GCs) response to oxidative stress, a common mechanism of environmental factors affecting female reproduction. We showed that nuclear miR-23a is a pro-apoptotic miRNA in porcine GCs through the activation of the transcription and function of *NORHA*, a long non-coding RNA (lncRNA) induces GC apoptosis and responses to oxidative stress. Mechanistically, miR-23a acts as an endogenous small activating RNA (saRNA) to alter histone modifications of the *NORHA* promoter through the direct binding to its core promoter. A C > T mutation was identified at −398 nt of the miR-23a core promoter, which created a novel binding site for the transcription factor SMAD4 and recruited the transcription repressor SMAD4 to inhibit miR-23a transcription and function in GCs. Notably, g.−398C > T mutation in the miR-23a promoter reduced GCs response to oxidative stress. In addition, g.−398C > T mutation was significantly associated with sow fertility traits. In short, our findings preliminarily revealed the genetic basis of individual differences in the response to oxidative stress from the perspective of a single mutation and identified miR-23a as a candidate gene for the environmental adaptation to oxidative stress.

## 1. Introduction

miRNAs are a class of small RNA molecules consisting of 21–25 nucleotides (nt) that are widely conserved in evolution. Since the discovery that lin-4, the first miRNA, controls temporal pattern formation in *Caenorhabditis elegans*, miRNAs have been proved to be involved in almost all physiological and pathological processes through a mechanism known as RNA interference (RNAi) [1,2]. Briefly, within the cytoplasm, mature miRNAs are first loaded into miRNA-induced silencing complexes (miRISCs), a ribonucleoprotein complex, and then bind to miRNA response elements (MREs) in the 3′ untranslated regions (UTRs) of mRNAs by base pairing, which in turn initiates mRNA decay or repress translation to post-transcriptionally repress target expression [3]. Notably, in addition to the cytoplasm, some mature miRNAs have recently been detected in the nucleus [4,5]. Interestingly, nuclear mature miRNAs can activate rather than repress the transcription of target mRNAs through a new mechanism termed RNA activation (RNAa) [6]. Briefly, nuclear mature miRNAs act as an endogenous small activating RNA (saRNA) directly interacting with MRE motifs in the promoter region of target mRNAs by base pairing in order to alter histone modifications and recruit RNA polymerase II, finally affecting the transcriptional activity of target mRNAs [6,7,8]. However, only a few miRNAs have been identified as controlling the transcription of target mRNAs through an RNAa mechanism.

Single nucleotide variants (SNVs) are one of the most common types of heritable variations. SNVs in the key protein-coding genes are usually associated with health and disease in humans [9,10] and with economically important traits in domestic animals [11,12] through altering the regulation and/or function of these genes. Although—compared with protein-coding genes—the miRNA sequence is shorter (pre-miRNAs are only about 80 bp in length) and there are less SNVs, their SNVs have also been shown to be involved in multiple important traits in humans and domestic animals through altering the expression and functions of target genes by influencing the biogenesis [13], transcription [14], and target specificity [15] of miRNAs. rs2291418 in pre-miR-1229, for instance, is a causal SNV for Alzheimer’s disease (AD) through the control of miR-1229-3p biogenesis and the repression of the translation of its direct target, *SORL1*, which encodes an AD-associated protein in the human brain [16]. Mechanistically, the SNV rs2291418 is conducive to the formation of a hairpin structure and breaks the equilibrium between the G-quadruplex structure and the hairpin structure in pre-miR-1229, thereby enhancing miR-1229-3p maturation [17].

As a multifunctional modulator and a member of the miR-23~24~27 cluster, miR-23a has been shown to induce granulosa cell (GC) apoptosis by targeting SMAD5, and it contributes to the ovarian function in women [18]. However, the role and mechanism of miR-23a in other mammalian ovaries and its relationship with female fertility are largely unknown. In this study, we showed that miR-23a plays a pro-apoptotic role in porcine GCs (pGCs) through the transactivation of *NORHA*, a pro-apoptotic lncRNA, by an RNAa mechanism. We also showed that a point mutation in the miR-23a promoter is a causal mutation for sow fertility traits through the prevention of GC apoptosis by recruiting the transcription factor SMAD4, the core component of the TGF-β signaling pathway, and reducing GCs response to oxidative stress by decreasing the level of *FoxO1*, an effector for oxidative stress.

## 2. Methods

### 2.1. Samples

A total of 346 Yorkshire sows with 3644 fertility trait records were randomly selected from the Jiangsu Kangle Breeding Farm (Changzhou, China) and were used for SNV screening and genotyping. Porcine ovaries were collected from the mature sows (180 days of age) of the local slaughterhouse and were used to isolate and culture pGCs. The experiments related to pigs were approved by the Animal Ethics Committee of Nanjing Agricultural University (SYXK (SU) 2015-0656).

### 2.2. Bioinformatic Analysis

miR-23a mature sequences were downloaded from miRbase (available online: https://www.mirbase.org/ (accessed on 1 October 2020)). The targets of miR-23a were forecast with miRDB (available online: http://mirdb.org/ (accessed on 22 May 2021)), Targetscan (available online: http://www.targetscan.org/vert_72/ (accessed on 22 May 2021)), and TarBase (available online: http://www.microrna.gr/tarbase (accessed on 22 May 2021)). The David tool (available online: https://david.ncifcrf.gov/ (accessed on 22 May 2021)) was carried out for the function enrichment analysis of the targets. JASPAR (available online: http://jaspar.genereg.net/ (accessed on 8 September 2021)) was used to forecast the binding sites for the transcription factors in the miR-23a promoter (the screening binding fraction was 90%). MREs in the *NORHA* promoter were predicted with RNAhybrid (available online: https://bibiserv.cebitec.uni-bielefeld.de/rnahybrid/ (accessed on 7 June 2021)).

### 2.3. Cell Culture, Transfection and Quantitative Real-Time PCR (qRT-PCR)

First, 3~5 mm (diameter) of healthy follicles were used to collect pGCs, and the pGCs were obtained and cultured, as mentioned earlier [19]. After 12 h of culture, DNA plasmids, miR-23a mimics, and an miR-23a inhibitor were transfected for a subsequent apoptosis assay, qRT-PCR, chromatin immunoprecipitation (ChIP), and a western blot assay by using HighGene (RM09014, ABclonal, Wuhan, China) according to the manufacturer’s protocol. All of the experiments were conducted in triplicate. The oligonucleotides are listed in Table S1. The total RNA was extracted with TRIzol (15596018, Invitrogen, Waltham, MA, USA), and its quality was checked by a microspectrophotometer (Thermo, Waltham, MA, USA), ensuring that the values of OD280/OD260 were between 1.9 and 2.0. Then, 500 ng of total RNA was reverse-transcribed into cDNA by using the HiScript II Q Select RT SuperMix (R232-01, Vazyme, Nanjing, China) according to the manufacturer’s protocol. The cDNA was diluted to appropriate concentrations for qRT-PCR on a StepOnePlus System (Applied Biosystems, Waltham, MA, USA) for a total of 40 cycles by using the SYBR Green Master Mix Kit (Q111-02/03, Vazyme, Nanjing, China). The reaction system was 10 μL, containing 0.2 μL each of the upstream and downstream primers (primer concentration 10 μmol/L) and 10% cDNA, which contained a denaturation temperature of 95 °C and an annealing temperature of 60 °C. Among them, the determination of primer efficiency and the PCR amplification efficiency was achieved by a series of dilutions of the cDNA template ranging from 95% to 105%, and the presence of a single peak in 80–90 °C confirmed the specificity of the PCR amplification product when the melting curve analysis was performed after qRT-PCR. *U6* was used as an internal reference for the miRNA, and *GAPDH* was used as an internal reference for the other genes [12]. Finally, the relative expression level was worked out by the 2^−ΔΔCT^ method. The primers for the RT-PCR are shown in Table S2.

### 2.4. Flow Cytometry

After transfection for 48 h, 5 × 10^5^ cells were harvested after trypsinization (25200072, Thermo, Waltham, MA, USA) and were washed twice with pre-chilled PBS. The staining was performed by adding 100 μL Binding Buffer, 5 μL annexin V, and 5 μL Propidium Iodide (PI) (A211-01/02, Vazyme, Nanjing, China) stain for 10 min. The samples were finally diluted by adding 400 μL Binding Buffer and were sequentially detected using a flow cytometer (Becton Dickinson, Franklin Lakes, NJ, USA). The results were analyzed using flowjo V7.6 software. The data from 10,000 cells were collected for each data file and analyzed by dividing the complete granule cell community using a cross shaped gating, guaranteeing the same gating settings between the treatment groups.

### 2.5. Subcellular Localization of miR-23a

First, 5 × 10^5^ pGCs were harvested after trypsinization and lysed in 500 μL lysis buffer (PBS, 0.1% NP-40, 10 mmol/L RNase inhibitor) for 10 min on ice. Then, the cells were centrifuged at 5000× *g* for 3 min at 4 °C. The supernatant was collected for cytoplasmic RNA extraction. The remaining particles were lysed and centrifuged again, and then the particles were collected again for nuclear RNA extraction. The nuclear and cytoplasmic RNA were validated using *U6* and *GAPDH*, respectively, and the nuclear and cytoplasmic RNA were used for the subcellular localization analysis of miR-23a using qRT-PCR.

### 2.6. Plasmid Construction and Dual-Luciferase Reporter Assays

The overexpression plasmids pcDNA3.1-*SMAD4* and pcDNA3.1-*NORHA* were previously constructed [19,20]. For the plasmid pcDNA3.1-*NR2C1*, the coding sequence (CDS) region of *NR2C1* was amplified from the cDNA of pGCs by using primers containing enzymatic digestion sites and cloned into a pcDNA3.1-basic vector (Promega, Madison, WI, USA). To construct luciferase reporters, the ssc-miR-23a promoter with four deletion fragments (−1446/−101, −1118/−101, −658/−101, and −289/−101) and different alleles for SNV was amplified from porcine genomic DNA by primers containing enzymatic digestion sites and cloned into a pGL3-basic vector (Promega, Madison, WI, USA). The vector and amplified products were digested with both *Nhe*I and *Xho*I for 2 h at 37 °C, purified, and ligated with T4 ligase (EL0016, Thermo, Waltham, MA, USA) at 16 °C overnight. The ligation products were transformed into competent cells (TSC-C01, Tsingke, Beijing, China) and plated, and single colonies to be grown were sequenced. Reporters of *NORHA* containing MRE-wt and MRE-mut were constructed by Tsingke (China). The primers are shown in Table S3. pGCs were assayed for luciferase activity using the dual-luciferase reporter assay system after 24 h of transfection (Promega, Madison, WI, USA).

### 2.7. ChIP

First, 1 × 10^7^ pGCs were cross-linked in 1% formaldehyde for 10 min and sonicated 13 times (10 s each) with a microneedle on ice with an output control setting of 30%. The chromatin solution was collected after the ultrasound and half was left as the positive control (input). Then, the remaining ultrasound treated chromatin was diluted 2.5 times and incubated with 40 protein A/G agarose at 4 °C for 1 h. The supernatant was then collected and incubated with an anti-SMAD4 antibody (D120124, Biotechnology), anti-H3K9me2 antibody (A2359, ABclonal), anti-H3K4me3 antibody (A2360, ABclonal), and anti-Ago2 antibody (A6023, ABclonal), respectively, at 4 °C overnight to pull down the DNA-protein complex. After de-crosslinking, DNA fragments were detected for enrichment by qRT-PCR. The antibody against IgG (D110058, Biotechnology) was taken as the internal control, and the unprocessed chromatin was taken as the input control. The primers for ChIP are shown in Table S4.

### 2.8. Western Blotting

After transfection for 48 h, 100 μL Ripa lysis buffer (AKR-191, AnnoRon, Beijing, China) was used to lyse the cells, and 10 min later, the cells were harvested with a cell scraper for the total protein. The protein concentration was determined using a concentration determination kit (#BL521A, Beyotime, Nantong, China), and a protein loading buffer (C516031, Sangon Biotech, Shanghai, China) was added for denaturation (100 °C, 10 min) after homogeneous sample concentration. Proteins of different sizes were separated by electrophoresis, transferred to PVDF membranes by transfer blotting, and blocked in 5% BSA for 2 h. The bands of interest were excised after the end of blocking, and the primary antibody was incubated overnight at 4 °C. At the end of the incubation, the strips were washed using TBST, and the secondary antibody was incubated for 1 h at room temperature. After washing the bands again, they were reacted in an ECL luminescent solution (E412-01, Vazyme, Nanjing, China) for 1–2 min, and protein detection was performed with a chemiluminescent gel imaging system (Bio-Rad, Hercules, CA, USA). The antibodies included anti-SMAD4 (D120124, Sangon Biotech, 1:1000), anti-NR2C1 (A6675, ABclonal, 1:1000), anti-*FoxO1* (D190665, Sangon Biotech, 1:1000), anti-GAPDH (TA802519, ORIGENE, 1:3000), and anti-β-Tubulin (10068-1-AP, Proteintech, 1:3000). β-Tubulin and GAPDH were measured as the internal control [12,21].

### 2.9. H_2_O_2_ Treatment

For the H_2_O_2_ treatment, the culture medium was first changed by DMEM/F12 without FBS for 6 h, and then H_2_O_2_ (Sigma, shanghai, China) was added into the medium with the final concentration at 200 μM for 2 h [21].

### 2.10. DNA Extraction, Genotyping, and Association Analysis

First, 20–40 mg ear tissue was digested with 200 μL DNA lysis buffer (100 mmol Tris-Hcl, 25 mmol EDTA, 500 mmol NaCl, and 1% SDS) containing protease K (100 μg/mL) at 55 °C for 12 h. The impurities were then removed by sequential saturation with Tris saturated phenols, chloroform, and isoamyl alcohol. The DNA was diluted to 50 ng/μL as a template for PCR amplification. The SNVs were screened using pooled-DNA sequencing and genotyped by DNA sequencing. The primers are shown in Table S5. The general linear model (GLM) of the SAS v9.2 software (SAS Institute, Cary, NC, USA) was used to analyze the correlation between the SNVs in miR-23a and the sow fertility traits, as described in our previous report [12].

### 2.11. Statistical Analysis

The data are presented as the mean ± S.E.M, and each experiment was carried out at least three biological replicates. The statistical analysis and graphing were performed using the GraphPad Prism v5 software (GraphPad Software, San Diego, CA, USA), and the significance was tested using a two-tailed *t*-test or a one-way ANOVA with a least significant difference test and a multiple comparisons test.

## 3. Results

### 3.1. miR-23a Is a Pro-Apoptotic miRNA in pGCs

**Figure 1:**
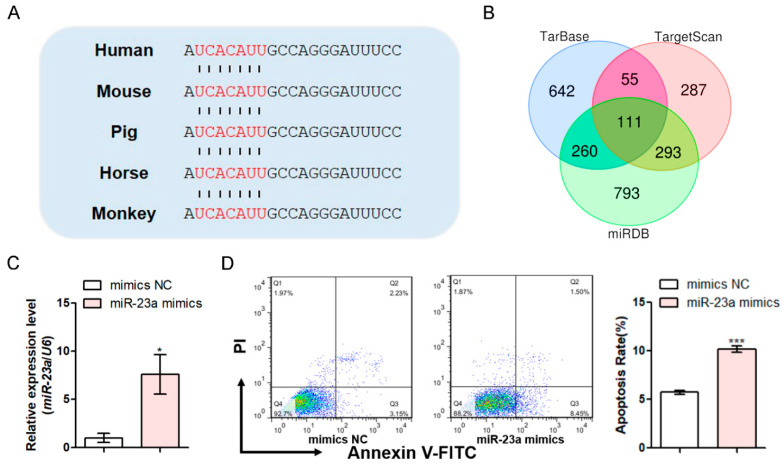

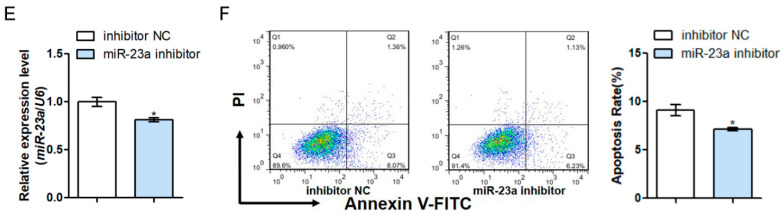
miR-23a induces pGC apoptosis. (**A**) Multiple alignments of the mature sequences of miR-23a in mammals. The bases marked in red are the seed sequence. (**B**) The prediction of potential targets for miR-23a was performed using TarBase (blue), TargtScan (red), and miRDB (green). (**C**,**D**) After transfection with miR-23a mimics, miR-23a levels were examined by qRT-PCR at 24 h (**C**), and the apoptosis rate was examined by fluorescence-activated cell sorting (FACS) at 48 h (**D**) in pGCs. (**E**,**F**) After transfection with the miR-23a inhibitor, miR-23a levels were examined by qRT-PCR at 24 h (**E**), and the apoptosis rate was examined by FACS at 48 h (**F**) in pGCs. Data are expressed as the mean ± S.E.M., and the significance was tested using a *t*-test. (* *p* < 0.05, *** *p* < 0.001).

The multi-sequence alignment showed that the porcine miR-23a gene shares high nucleotide identities with other mammals (Figure S1). Furthermore, both the mature and seed sequences of miR-23a are completely conserved in mammals including pigs (Figure 1A). To understand the potential function of miR-23a, a total of 111 putative targets were predicted using three bioinformatics tools—TargetScan, TarBase, and miRDB (Figure 1B and Table S6)—which were mainly distributed in biological processes and pathways related to cancer, transcriptional regulation, and apoptosis (Figure S2). Combined with previous studies in hGC apoptosis [18], we next focused on the role of miR-23a in pGC apoptosis. The flow cytometry showed that there was a greater induction of the apoptotic rate of pGCs when it was transfected with miR-23a mimics (an endogenous analog of miR-23a) compared to the transfection of mimics NC (nonsense scrambled) (Figure 1C,D), whereas the opposite was observed when it was transfected with miR-23a inhibitor (a suppressor of miR-23a) compared to transfection of inhibitor NC (nonsense scrambled) (Figure 1E,F). These results suggest that miR-23a is a pro-apoptotic miRNA in the GCs of the porcine ovary.

### 3.2. miR-23a Activates the Transcription and Function of lncRNA NORHA

**Figure 2:**
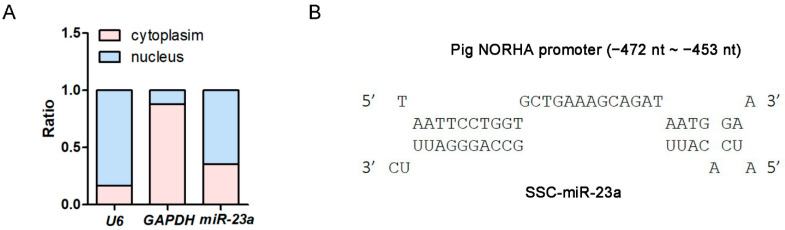

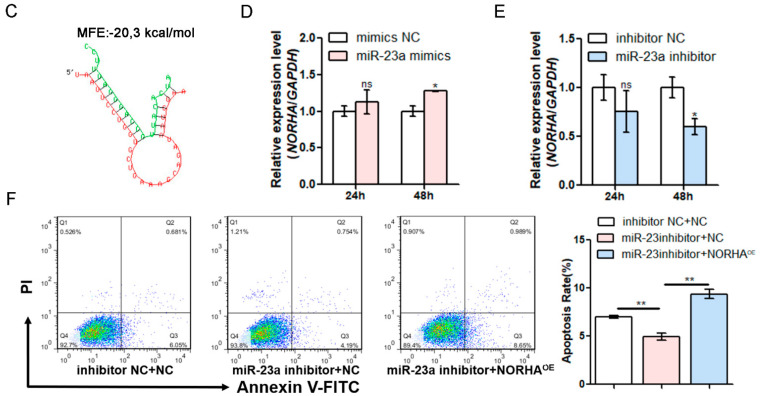
miR-23a induces pGC apoptosis by activating *NORHA* expression. (**A**) Subcellular localization analysis of miR-23a by nucleocytoplasmic separation. *U6* is a reference for the nucleus (blue) and *GAPDH* is a reference for the cytoplasm (red). (**B**,**C**) Potential binding of miR-23a to the lncRNA *NORHA* promoter. (**B**) An MRE of miR-23a was located in the *NORHA* promoter. (**C**) The minimum free energy (MFE) was predicted by RNAhybrid. (**D**,**E**) After transfection with miR-23a mimics (**D**) or the miR-23a inhibitor (**E**), *NORHA* levels were examined by qRT-PCR at 24 h and 48 h in pGCs. (**F**) After co-transfection with the miR-23a inhibitor and pcDNA3.1-*NORHA*, the pGC apoptosis rate was examined by FACS at 48 h. Data are expressed as the mean ± S.E.M., and significance was tested using a *t*-test (**D**,**E**) and a one-way ANOVA (**F**) (ns, not significant, * *p* < 0.05, ** *p* < 0.01).

Nuclear and cytoplasmic miRNAs have been shown to control cell functions by different mechanisms (RNAi or RNAa) [22]. We performed a subcellular localization assay by separating nuclear and cytoplasmic RNA and showed that miR-23a is more enriched in the nucleus than it is in the cytoplasm of pGCs (Figure 2A), indicating that miR-23a induces pGC apoptosis, possibly via an RNAa mechanism. Interestingly, an MRE motif was detected in the promoter region of the porcine *NORHA*, a pro-apoptotic lncRNA in pGCs (Figure 2B), when we previously analyzed its promoter sequence [19]. Furthermore, the two have a strong potential interaction ability with the -20 kcal/mol of the minimum free energy (MFE) (Figure 2C), so NORHA was selected for further investigation. As expected, we noted that the overexpression of miR-23a significantly activates *NORHA* transcription in pGCs at 48 h, but not at 24 h (Figure 2D), which is in line with the first feature of the RNAa mechanism: delayed action time. Similarly, the knocking down of miR-23a significantly inactivates *NORHA* transcription in pGCs at 48 h, but not at 24 h (Figure 2E). To investigate whether miR-23a induces pGC apoptosis through the activation of *NORHA* transcription, both the miR-23a inhibitor and pcDNA3.1-*NORHA* were co-transfected into pGCs cultured in vitro. The overexpression of *NORHA* significantly reversed the reduction of the pGC apoptosis rate caused by the miR-23a inhibitor (Figure 2F). In summary, these data suggest that miR-23a enhances pGC apoptosis through the activation of *NORHA* transcription.

### 3.3. miR-23a Is a saRNA of lncRNA NORHA

**Figure 3:**
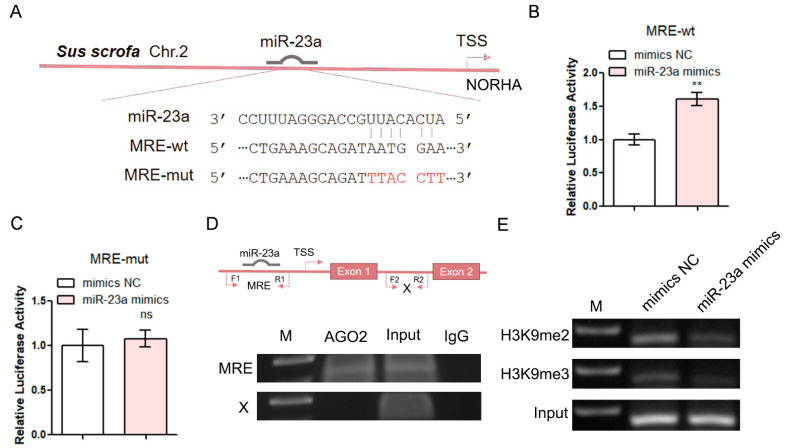
miR-23a enhances *NORHA* promoter activity by altering histone modification of the MRE motif. (**A**) Vector diagram of the wild type (wt) and mutated type (mt) of the MRE motif in the NORHA promoter. The red base is the mutation site. TSS, transcription start site. (**B**,**C**) Luciferase activity was detected in pGCs after being co-transfected with miR-23a mimics and a reporter vector containing the MRE-wt (**B**) or the MRE-mut (**C**). (**D**) ChIP assays were performed using an AGO2-specific antibody. The image contains a schematic diagram of the porcine *NORHA* gene structure (top) and ChIP images (bottom). F1/R1 and F2/R2 are the primers used to amplify the MRE site and X site, respectively. The X site is an intronic region of *NORHA* that does not contain the MRE of miR-23a. M, DNA marker. (**E**) ChIP assays were performed using H3K9me2- and H3K9me3-specific antibodies. Data are expressed as the mean ± S.E.M., and significance was tested using a *t*-test (ns, not significant, ** *p* < 0.01).

To test the miR-23a activation of lncRNA *NORHA* transcription via miR-23a MRE in its promoter, two reporter vectors of the porcine *NORHA* promoter were generated (Figure 3A). The luciferase reporter assays showed that the luciferase activities of the *NORHA* promoter were significantly increased upon the overexpression of miR-23a in pGCs, while no significant changes were observed in the luciferase activities of the promoter with a mutation-type MRE motif of miR-23a (Figure 3B,C), indicating that miR-23a enhances the promoter activity of *NORHA* via an MRE motif of miR-23a in its promoter. The activation of target transcription by saRNAs has been shown to depend on AGO2 [23]. We therefore performed a ChIP assay by a specific anti-AGO2 antibody to assess whether miR-23a guides AGO2 to bind to the *NORHA* promoter in pGCs. Compared with the X site (without the MRE motif of miR-23a) within the intron 1 of *NORHA*, a significant enrichment of AGO2 binding was observed in the MRE motif of miR-23a in the region of the *NORHA* promoter (Figure 3D), indicating that AGO2 binding was guided by miR-23a. In addition, RNAa has also been shown to be involved in the histone modification at the target promoter region [22,24]. As expected, the overexpression of miR-23a resulted in the decreased enrichment of H3K9me2 and H3K9me3 at the MRE motif for miR-23a in the region of the *NORHA* promoter (Figure 3E). Overall, these data suggest that miR-23a is a saRNA of lncRNA *NORHA* in pGCs.

### 3.4. g.−398C > T Mutation in the miR-23a Promoter Decreases Its Transcriptional Activity

**Figure 4:**
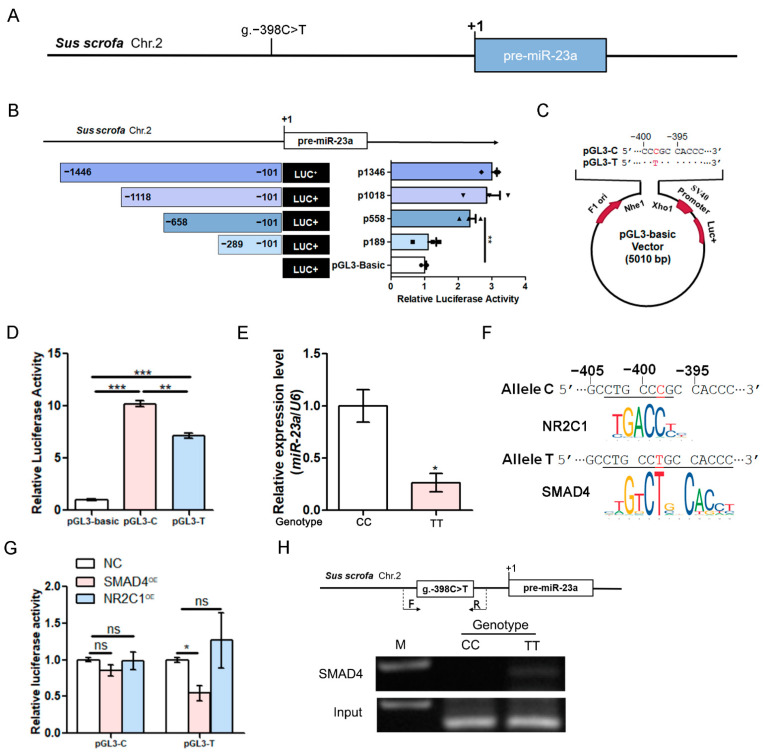
The SNV g.−398C > T affects *miR-23a* promoter activity by recruiting the transcription factor SMAD4. (**A**) A point variant C > T was detected at −398 nt in the *miR-23a* promoter. (**B**) Identification of the core promoter region of miR-23a. The image contains a schematic representation of the four deletion constructions (left) and their luciferase activity (right). The first base of pre-miR-23a was taken as +1. (**C**) Schematic showing the constructions of pGL3-C and pGL3-T. (**D**) Reporter activity assays. pGL3-C and pGL3-T were transfected into pGCs; luciferase activity was detected. (**E**) miR-23a levels were determined in pGCs with different genotypes for the SNV g.−398C > T. GCs were isolated from the ovaries of sows with genotype CC (*n* = 3) and genotype *TT* (*n =* 3). (**F**) Prediction of binding sites for transcription factors in the *miR-23a* promoter with allele C and allele T by JASPAR. (**G**) Effects of transcription factors SMAD4 and NR2C1 on the activity of the miR-23a promoter with allele *C* and allele *T*. (**H**) ChIP assay. The image contains a schematic diagram of the porcine miR-23a (top) and ChIP images (bottom). F/R are the primers used to amplify a DNA fragment containing the SMAD4 binding element (SBE) motif. Data are expressed as the mean ± S.E.M., and significance was tested using a *t*-test (**B**,**E**) and a one-way ANOVA (**D**,**G**) (ns, not significant, * *p* < 0.05, ** *p* < 0.01, *** *p* < 0.001).

Interestingly, a C/T point mutation was detected at −398 nt of the promoter region of the porcine miR-23a gene, which was named g.−398C > T (Figure 4A). To investigate whether the SNV g.−398C > T is located in the core promoter of the porcine miR-23a gene, we identified its core promoter by luciferase reporter assays using deletion constructs. As shown in Figure 4B, a DNA region from −658 to −289 nt is the core promoter of the porcine miR-23a gene, and the SNV g.−398C > T was located in this region. To further determine whether the SNV g.−398C > T affects miR-23a promoter activity, two reporter vectors of the core promoter with alleles C and T were constructed (Figure 4C). The luciferase activities of the reporter vector with allele C were significantly higher than that with allele T in the pGCs (Figure 4D). Furthermore, the expression levels of miR-23a in the pGCs of sows with genotype CC were significantly higher than those with genotype TT (Figure 4E). In general, these data suggest that the SNV g.−398C > T is strongly involved in the transcriptional activity of miR-23a in pGCs through the influence of its promoter activity.

### 3.5. g.−398C > T Mutation in the miR-23a Promoter Creates a Novel Binding Site for the Transcription Factor SMAD4

To investigate the mechanism by which the SNV g.−398C > T decreases miR-23a promoter activity, we predicted the binding sites for the transcription factors in its core promoter. Notably, a putative binding site for the transcription factor NR2C1 was only detected in the miR-23a promoter with allele C for the SNV g.−398C > T, and a putative binding site for the transcription factor SMAD4 was only detected in the miR-23a promoter with allele T for the SNV g.−398C > T (Figure 4F). The luciferase reporter assays showed that the transcription factor SMAD4 significantly reduces the activities of the miR-23a promoter with allele T, but not that with allele C (Figure 4G and Figure S3A). However, the transcription factor NR2C1 has not affected the activities of the miR-23a promoter with both allele C and allele T (Figure 4G and Figure S3B). The ChIP experiments showed that the transcription factor SMAD4 directly binds to the miR-23a promoter in the GCs from sows with genotype TT for the SNV g.−398C > T, but not those with genotype CC (Figure 4H). These results suggest that the SNV g.−398C > T produces a binding site for the transcription factor SMAD4, which decreases the transcriptional activity of miR-23a through recruiting the transcription factor SMAD4.

### 3.6. The SNV g.−398C > T Affects the SMAD4 Inhibition of miR-23a Expression

**Figure 5:**
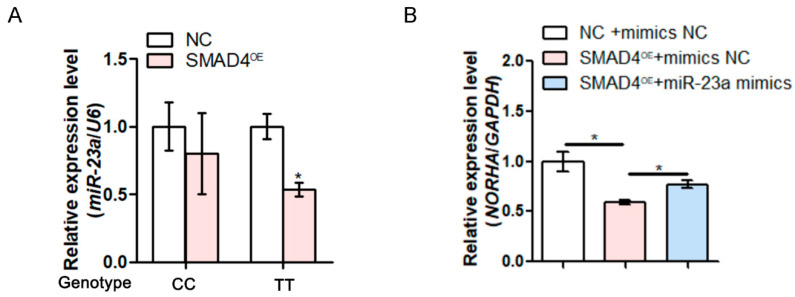

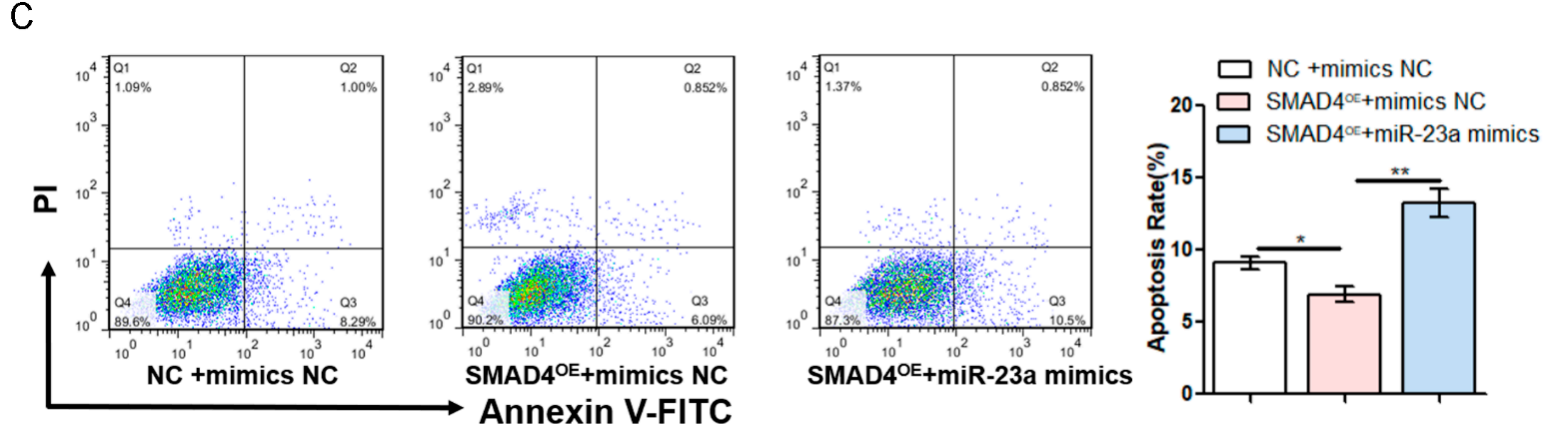
SMAD4 inhibits the expression and function of miR-23a. (**A**) After transfection with pcDNA3.1-SMAD4, miR-23a levels were detected in pGCs with genotypes CC and TT for the SNV g.−398C > T. (**B**,**C**) *NORHA* levels (**B**) and the apoptosis rate (**C**) in pGCs after co-transfection with pcDNA3.1-SMAD4 and miR-23a mimics at 48 h. Data are expressed as the mean ± S.E.M., and significance was tested using a *t*-test (**A**) and a one-way ANOVA (**B**,**C**) (ns, not significant, * *p* < 0.05, ** *p* < 0.01).

To investigate whether SMAD4 regulates miR-23a expression, we isolated the GCs from sows with different genotypes for the SNV g.−398C > T and treated them with pcDNA3.1-SMAD4. The overexpression of SMAD4 significantly inhibited the expression of miR-23a in the pGCs with genotype TT, but not those with genotype CC (Figure 5A), indicating that SMAD4 is a transcription repressor of miR-23a in the pGCs with genotype TT for the SNV g.−398C > T. Furthermore, a co-transfection experiment with pcDNA3.1-SMAD4 and miR-23a mimics showed that SMAD4 also significantly reduces the expression of *NORHA*, a direct target of miR-23a, while miR-23a can rescue this situation in pGCs (Figure 5B). Importantly, SMAD4 is an anti-apoptotic transcription factor in pGCs [20]. We therefore investigated whether miR-23a mediates the anti-apoptotic effect of SMAD4. Flow cytometry confirmed that SMAD4 significantly reduces the apoptosis rate of pGCs, while the overexpression of miR-23a can reverse the anti-apoptotic influence of SMAD4 in pGCs (Figure 5C,D). The above data suggest that SMAD4 is embroiled in miR-23a functions, including suppressing the downstream target *NORHA* expression, as well as the pGC apoptosis, via the regulation of miR-23a.

### 3.7. The miR-23a SNV g.−398C > T Affects the Resistance of GCs to Oxidative Stress

**Figure 6:**
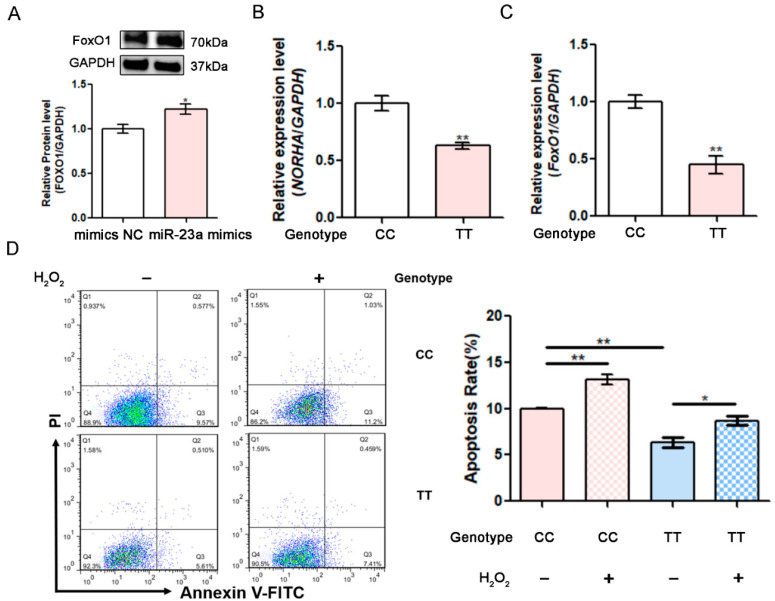
The miR-23a SNV g.−398C > T affects the response of pGCs to oxidative stress. (**A**) After transfection with miR-23a mimics, *FoxO1* protein levels were detected by western blotting in pGCs. (**B**,**C**) *NORHA* and *FoxO1* levels were determined in pGCs from the ovaries of sows with genotype CC (*n* = 3) and genotype TT (*n* = 3). (**D**) The apoptosis rate was detected by FACS in pGCs at 2 h after being treated with 200 μM H_2_O_2_. Data are expressed as the mean ± S.E.M., and significance was tested using a *t*-test. (* *p* < 0.05, ** *p* < 0.01).

Our previous study demonstrated that *FoxO1*, an effector for oxidative stress, is downstream of *NORHA* in pGCs [19]. Western blotting showed that the overexpression of miR-23a significantly induces the *FoxO1* protein level in pGCs (Figure 6A). Notably, the mRNA expression of *NORHA* and *FoxO1* in pGCs with genotype TT for the SNV g.−398C > T were significantly lower than that in pGCs with genotype CC (Figure 6B–C). To investigate whether the miR-23a SNV g.−398C > T influences the pGCs response to oxidative stress, we induced oxidative stress in pGCs by H_2_O_2_ exposure. Flow cytometry showed that pGCs with genotype TT for the SNV g.−398C > T have a significantly lower apoptosis rate than those with genotype CC (Figure 6D). This is consistent with the low expression of the pro-apoptotic factor miR-23a, *NORHA,* and *FoxO1* in pGCs with genotype TT for the SNV g.−398C > T. Interestingly, under H_2_O_2_ exposure, the increase in the apoptosis rate of pGCs with genotype TT for the SNV g.−398C > T was also lower than that of genotype *CC* (Figure 6D), indicating that pGCs with genotype *TT* for the SNV g.−398C > T are protected against oxidative stress relative to those with genotype CC. These data suggest that the miR-23a SNV g.−398C > T affects the response of pGCs to oxidative stress.

### 3.8. The miR-23a SNV g.−398C > T is Significantly Associated with Sow Fertility Traits

**Figure 7:**
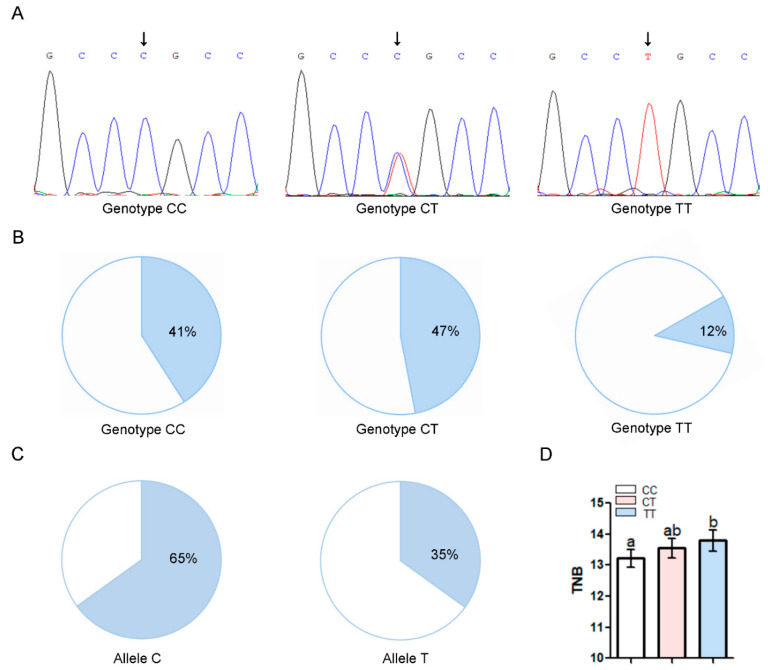
The SNV g. −398C > T within the miR-23a core promoter is associated with sow feritility traits. (**A**) Peak plot of g.−398C > T. (**B**) Genotype frequency of the SNV g.−398C > T in a Large White sow population (*n* = 346). (**C**) Allele frequency of the SNV g.−398C > T in a Large White sow population (*n* = 346). (**D**) Association analysis between the SNV g.−398C > T and TNB of sows for multiparities. Data are expressed as the least-squares mean ± S.E.M., *n* = 346, and significance was tested using a GLM program. Significant differences between groups (*p* < 0.05) are indicated by different lowercase letters, and insignificant differences (*p* > 0.05) are indicated by the same letter.

To investigate whether the SNV g.−398C > T in the miR-23a promoter is associated with sow fertility traits, the SNV g.−398C > T was genotyped in a Yorkshire sow population (*n* = 346) by direct sequencing. Three genotypes (CC, CT, and TT) with frequencies of 0.41, 0.47, and 0.12, respectively, were observed (Figure 7A,B). Moreover, the allele frequencies were 0.65 (allele C) and 0.375 (allele T) (Figure 7C), indicating that allele C is the dominant allele. To determine the effect of the SNV g.−398C > T on the fertility traits in Yorkshire sows, an association analysis was carried out using a mixed model. For multiparity, the total number of piglets born (TNB) of sows with genotype TT was significantly higher than that of those with genotype CC (Figure 7D). However, there is no significant difference in the other fertility traits, including the total number of piglets born alive (NBA), the number of stillbirths (NSB), and the litter weight (LW) among three genotypes (CC, CT, and TT) of the SNV g.−398C > T in this Yorkshire population (Figure S4). In summary, these results indicate that the miR-23a SNV g.−398C > T is significantly associated with sow fertility traits, and miR-23a is a candidate miRNA for sow fertility traits in the Yorkshire population.

## 4. Discussion

In porcine folliculogenesis, more than 95% of follicles will undergo atresia, which severely limits sow fertility. Endogenous miRNAs are strongly involved in porcine follicular atresia and control key characteristics during follicular atresia, such as miR-144 [25] and miR-2337 [22] control pGC apoptosis, the main causes of follicular atresia. Besides, the balance of progesterone (P4)/17β-estradiol (E2) concentration in follicular fluid, a core classification index of atretic follicles, is also modulated by miRNAs including miR-1275 [26] and miR-31-5p [27]. In this study, we identified miR-23a as a novel miRNA that induces pGC apoptosis in pigs. Previous studies showed that miR-23a promotes cell apoptosis in hGCs via the FasL-Fas, ERK1/2, and CDC42/PAK1 signaling pathways, which are related to ovarian reserve function and premature ovarian failure [18,28,29]. In hGCs, miR-23a has also been shown to block the cell cycle on the G0/G1 phase and suppress cell proliferation [28]. In addition to GCs, miR-23a is also an important regulator in many cell types such as human acute lymphoblastic T-cells [30], airway epithelial cells and fibroblasts [31], and oral squamous cell carcinoma cells [32]. In summary, our findings not only extend the function of miR-23a-regulating GC apoptosis from humans to domestic animals, they once again confirm the conservation of the function of miRNAs in the same cell types among different species and also provide a new potential non-hormonal drug for sow reproductive regulation.

lncRNAs and miRNAs are the two most studied endogenous non-coding RNAs (ncRNAs), which form a complex regulatory network with protein-coding genes through various forms of dialogue mechanisms to control health and disease. LncRNAs, for instance, (i) act as a molecular sponge of miRNAs [19], (ii) compete for protein-coding genes with miRNAs [33], (iii) act as a precursor or host of miRNAs [34], and (iv) contribute to miRNA biogenesis [35]. Meanwhile, miRNAs also inhibit lncRNA expression via an RNAi mechanism, just as they inhibit the 3′-UTRs of protein-encoding genes [36]. In this study, we identified a novel mechanism by which miRNAs regulate lncRNA—miR-23a acts as an endogenous small saRNA to promote the transcription of lncRNA *NORHA* through direct binding to the promoter of lncRNA *NORHA*. saRNAs are a kind of small double-stranded RNA (dsRNA) with a length of 19-21 nt which target the promoter regions of genes and induce gene transcription via an RNAa mechanism [6]. In the mid 2000s, Li et al. [24] found that chemically synthesized exogenous saRNA activates the transcription of target genes in mammalian cells. Subsequently, some exogenous saRNAs such as the *CEBPA*-saRNA targets *CEBPA* [37] and the saRNA-158 targets *VMP1* [38] have been identified. Since the mature sequence of miRNAs is similar to that of saRNAs in terms of length, scientists attempt to assess their feasibility as endogenous saRNAs [39]. In recent years, several miRNAs including miR-4281 [40], miR-320 [41], and miR-2337 [22] have been identified as the endogenous saRNAs target important functional genes. In conclusion, our findings prove for the first time that miRNA can serve as the saRNA of lncRNA, which establishes a new form of connection between miRNAs and lncRNAs. At the same time, our study also provides a theoretical basis for using miRNAs as endogenous saRNAs in order to enhance the transcriptional activation of favorable genes for female reproduction and improve female fertility.

Variants in the promoter region are strongly involved in gene transcription [42]. One of the important mechanisms is to affect the binding with transcription factors by creating a new transcription factor binding site or by losing an existing transcription factor binding site [43]. In this study, we identified, for the first time, the SNV g.−398C > T in the miR-23a promoter and showed that a new binding site for the transcription factor SMAD4 in the miR-23a promoter was created by the SNV g.−398C > T, which significantly inhibits the transcriptional activity and expression of miR-23a in pGCs with genotype TT, thereby reducing pGC apoptosis and increasing sow fecundity. As an important effector of the TGF-β signaling pathway and an anti-apoptotic factor in mammalian GCs, SMAD4 is well known to be involved in female reproduction, such as the fate (maturation or atresia) of follicular development [20], ovulation [44], and fertility [45]. Furthermore, SMAD4 generally functions as a transcription factor, directly acting on the key genes including the miRNAs of important events in the female reproductive cycle [46]. For example, the key genes *HAS2*, for inducing ovulation [47], and *ALK3*, for maintaining embryo implantation [48], are all transcriptional targets of SMAD4. Recently, several miRNAs involved in critical events in the female reproductive cycle, such as miR-29c, for influencing the follicular development fate [12], have also been shown to be directly regulated by the transcription factor SMAD4. Taken together, our findings not only identify an miRNA affecting GC apoptosis but also reveal a molecular mechanism by which the miR-23a SNV g.−398C > T affects miR-23a transcription and GC apoptosis. Meanwhile, our findings also define a new signaling pathway, SMAD4/miR-23a/*NORHA*, affecting GC apoptosis, which provides new insights into the mechanism of GC apoptosis and female fertility in mammals.

Oxidative stress is a major trigger of follicular atresia and GC apoptosis which impairs follicular development, leads to a variety of ovarian diseases, and limits the fertility of female animals including pigs [49,50,51]. *FoxO1* is a major sensor and effector of oxidative stress [52] which can promote cell apoptosis in response to oxidative stress by activating downstream apoptosis-related proteins such as Bim [53], P21 [54], and Fasl [55]. Similarly, it has also been reported that inhibiting *FoxO1* by different means can effectively block the effect of oxidative stress [56]. Here, we found that the level of *FoxO1*, downstream of *NORHA* (a direct target of miR-23a), was decreased in pGCs with genotype TT for the miR-23a SNV g.−398C > T, indicating that the SNV g.−398C > T may affect the response of pGCs to oxidative stress. Subsequently, this hypothesis was confirmed by an in vitro oxidative stress model for pGCs. Multiple environmental stresses in swine production often cause the elevation of oxidative free radicals between the organism and tissues, so oxidative stress is a common mechanism by which multiple stressors impair reproductive performance [57]. At present, there are many studies on the mechanisms by which oxidative stress affects reproductive performance in sows, but there are few studies on the differentiation of sows in response to oxidative stress and the underlying mechanisms [58]. In addition, the growing practice suggests that differences between the rearing environment of breeding pigs (friendly environment) and that of commercial pigs (a more challenging environment) result in some individuals often failing to exhibit their due genetic potential, and, therefore, genotype-by-environment interactions (GEI) are increasingly being considered in pig breeding programs in order to improve the environmental adaptation, productivity, and economic benefits for the pig industry [59,60,61]. Therefore, GEI is becoming an important focus for the pig industry. In conclusion, our findings reveal that a mechanism underlying mutation affects sow fertility by exerting different responses to the external environment, which provide a potential molecular marker for selecting sows with high environmental adaptation and fertility. Meanwhile, our findings also shed new light on the molecular mechanism of GEI in regulating economically important traits (i.e., reproductive and growth traits) in livestock and the personalized regulation of sow reproduction.

miRNAs are required for female reproduction and are involved in all the important events in the mammalian reproductive cycle from the establishment of the primordial follicle pool to pregnancy and parturition [62]. The knockout of key genes such as *Dicer* and *Drosha/DGCR8* in the miRNA biogenesis pathway can lead to changes in the female fertility of mice [63]. Indeed, a significant association between miR-604A>G and miR-938G>A polymorphism and idiopathic recurrent pregnancy loss were observed in Korean women [64]. In sows, miRNAs can control the fate of follicular development, and their levels are involved in sow fertility [65,66]. However, no candidate miRNAs for sow fertility traits have been identified. Although two SNVs (g.−573G>A and g.−40T>C) were identified in the miR-130a promoter region by our group, they were not associated with the fertility traits in a Yorkshire sow population [67]. In addition, a *T > C* variant has been revealed to be associated with the TNB and NBA traits in a DIV sow population; however, it is located at 18 nt downstream of the pre-miR-27a [65]. In this study, we showed that the SNV g.−398C > T in the miR-23a promoter is associated with the TNB trait, demonstrating that miR-23a is a candidate miRNA for the fertility traits of Yorkshire sows. Together, our findings identified, for the first time, a causal miRNA for sow fertility traits, which provides a potential genetic marker for molecular breeding in Yorkshire sows and confirms that miRNA can serve as a new carrier for the genetic markers of sow fertility traits. However, this candidate genetic marker remains to be verified in pig breeding.

## 5. Conclusions

**Figure 8:**
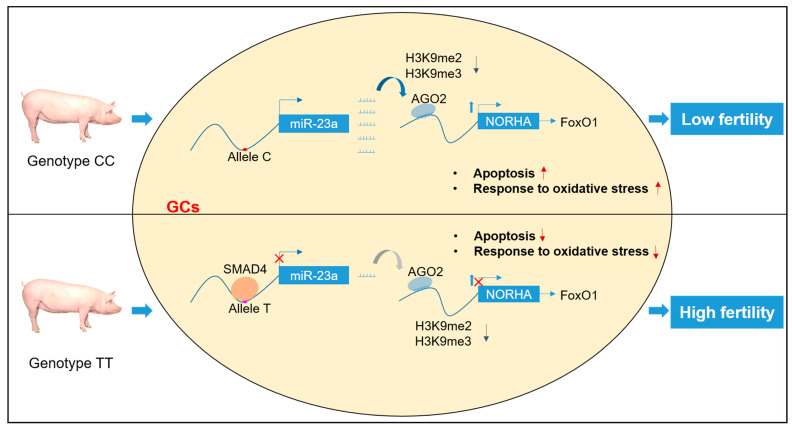
Working model. The SNV g.−398C > T for sow fertility traits in the miR-23a promoter mediated the regulation of *NORHA* expression by affecting miR-23a expression, which in turn affected pGCs apoptosis and the pGCs response to oxidative stress.

In conclusion, we demonstrated that miR-23a acts as a saRNA to activate lncRNA *NORHA* transcription in pGCs through the alteration of histone modifications in the promoter region by directly binding to its promoter in order to induce GC apoptosis. Interestingly, we identified the SNV g.−398C > T in the miR-23a promoter, which is strongly involved in miR-23a transcription and functions by recruiting the transcription repressor SMAD4. Importantly, this SNV also causes a decrease in the sensitivity of GCs to oxidative stress, thereby increasing sow fertility (Figure 8). Our findings provide an endogenous saRNA for regulating sow reproduction and potential molecular markers for breeding sows with strong environmental adaptability and high fecundity.

## Data Availability

Data is contained within the article.

## Supplementary Materials

The following supporting information can be downloaded at: https://www.mdpi.com/article/10.3390/antiox11061174/s1.

## References

[1] Bartel D.P. (2018). Metazoan microRNAs. Cell.

[2] Krol J., Loedige I., Filipowicz W. (2010). The widespread regulation of microRNA biogenesis, function and decay. Nat. Rev. Genet..

[3] Wilczynska A., Bushell M. (2015). The complexity of miRNA-mediated repression. Cell Death Differ..

[4] Politz J.C., Hogan E.M., Pederson T. (2009). MicroRNAs with a nucleolar location. RNA.

[5] Wong J.J., Ritchie W., Gao D., Lau K.A., Gonzalez M., Choudhary A., Taft R.J., Rasko J.E., Holst J. (2014). Identification of nuclear-enriched miRNAs during mouse granulopoiesis. J. Hematol. Oncol..

[6] Ghanbarian H., Aghamiri S., Eftekhary M., Wagner N., Wagner K.D. (2021). Small Activating RNAs: Towards the development of new therapeutic agents and clinical treatments. Cells.

[7] Barlak N., Capik O., Kilic A., Sanli F., Aytatli A., Yazici A., Karatas E.A., Ortucu S., Karatas O.F. (2021). MicroRNA-145 transcriptionally regulates Semaphorin 3A expression in prostate cancer cells. Cell Biol. Int..

[8] Huang V., Place R.F., Portnoy V., Wang J., Qi Z., Jia Z., Yu A., Shuman M., Yu J., Li L.C. (2012). Upregulation of Cyclin B1 by miRNA and its implications in cancer. Nucleic Acids Res..

[9] Findlay G.M., Daza R.M., Martin B., Zhang M.D., Leith A.P., Gasperini M., Janizek J.D., Huang X., Starita L.M., Shendure J. (2018). Accurate classification of BRCA1 variants with saturation genome editing. Nature.

[10] Zhang Z., Liu X., Li L., Yang Y., Yang J., Wang Y., Wu J., Wu X., Shan L., Pei F. (2021). SNP rs4971059 predisposes to breast carcinogenesis and chemoresistance via TRIM46-mediated HDAC1 degradation. EMBO J..

[11] Clop A., Marcq F., Takeda H., Pirottin D., Tordoir X., Bibe B., Bouix J., Caiment F., Elsen J.M., Eychenne F. (2006). A mutation creating a potential illegitimate microRNA target site in the myostatin gene affects muscularity in sheep. Nat. Genet..

[12] Du X., Liu L., Wu W., Li P., Pan Z., Zhang L., Liu J., Li Q. (2020). SMARCA2 is regulated by NORFA-miR-29c, a novel pathway that controls granulosa cell apoptosis and is related to female fertility. J. Cell Sci..

[13] Le C., Nguyen T.L., Nguyen T.D., Nguyen T.A. (2020). Human disease-associated single nucleotide polymorphism changes the orientation of DROSHA on pri-mir-146a. RNA.

[14] Hou G., Harley I., Lu X., Zhou T., Xu N., Yao C., Qin Y., Ouyang Y., Ma J., Zhu X. (2021). SLE non-coding genetic risk variant determines the epigenetic dysfunction of an immune cell specific enhancer that controls disease-critical microRNA expression. Nat. Commun..

[15] Hill C.G., Jabbari N., Matyunina L.V., McDonald J.F. (2014). Functional and evolutionary significance of human microRNA seed region mutations. PLoS ONE.

[16] Ghanbari M., Ikram M.A., de Looper H., Hofman A., Erkeland S.J., Franco O.H., Dehghan A. (2016). Genome-wide identification of microRNA-related variants associated with risk of Alzheimer’s disease. Sci. Rep..

[17] Imperatore J.A., Then M.L., McDougal K.B., Mihailescu M.R. (2020). Characterization of a G-Quadruplex structure in pre-miRNA-1229 and in its Alzheimer’s disease-associated variant rs2291418: Implications for miRNA-1229 maturation. Int. J. Mol. Sci..

[18] Nie M., Yu S., Peng S., Fang Y., Wang H., Yang X. (2015). miR-23a and miR-27a promote human granulosa cell apoptosis by targeting SMAD5. Biol. Reprod..

[19] Yao W., Pan Z., Du X., Zhang J., Liu H., Li Q. (2021). NORHA, a novel follicular atresia-related lncRNA, promotes porcine granulosa cell apoptosis via the miR-183-96-182 cluster and FoxO1 axis. J. Anim. Sci. Biotechnol..

[20] Liu J., Du X., Zhou J., Pan Z., Liu H., Li Q. (2014). MicroRNA-26b functions as a proapoptotic factor in porcine follicular Granulosa cells by targeting Sma-and Mad-related protein 4. Biol. Reprod..

[21] Li X., Lin Y., Yao J., Pan B., Zhan X., Chen Z., Bai Y., Zhang H., Wang B., Chen S. (2021). Protegrin-1 inhibits porcine ovarian granulosa cell apoptosis from H2O2-induced oxidative stress via the PERK/eIF2α/CHOP signaling pathway in vitro. Theriogenology.

[22] Wang L., Du X., Li Q., Wu W., Pan Z., Li Q. (2021). miR-2337 induces TGF-beta1 production in granulosa cells by acting as an endogenous small activating RNA. Cell Death Discov..

[23] Meng X., Jiang Q., Chang N., Wang X., Liu C., Xiong J., Cao H., Liang Z. (2016). Small activating RNA binds to the genomic target site in a seed-region-dependent manner. Nucleic Acids Res..

[24] Li L.C., Okino S.T., Zhao H., Pookot D., Place R.F., Urakami S., Enokida H., Dahiya R. (2006). Small dsRNAs induce transcriptional activation in human cells. Proc. Natl. Acad. Sci. USA.

[25] Zhou J., Lei B., Li H., Zhu L., Wang L., Tao H., Mei S., Li F. (2017). MicroRNA-144 is regulated by CP2 and decreases COX-2 expression and PGE2 production in mouse ovarian granulosa cells. Cell Death Dis..

[26] Liu J., Li X., Yao Y., Li Q., Pan Z., Li Q. (2018). miR-1275 controls granulosa cell apoptosis and estradiol synthesis by impairing LRH-1/CYP19A1 axis. Biochim. Biophys. Acta Gene Regul. Mech..

[27] Yuan C., Li Z., Zhao Y., Wang X., Chen L., Zhao Z., Cao M., Chen T., Iqbal T., Zhang B. (2021). Follicular fluid exosomes: Important modulator in proliferation and steroid synthesis of porcine granulosa cells. FASEB J..

[28] Lin J., Huang H., Lin L., Li W., Huang J. (2020). MiR-23a induced the activation of CDC42/PAK1 pathway and cell cycle arrest in human cov434 cells by targeting FGD4. J. Ovarian Res..

[29] Luo H., Han Y., Liu J., Zhang Y. (2019). Identification of microRNAs in granulosa cells from patients with different levels of ovarian reserve function and the potential regulatory function of miR-23a in granulosa cell apoptosis. Gene.

[30] Morey T.M., Roufayel R., Johnston D.S., Fletcher A.S., Mosser D.D. (2015). Heat shock inhibition of CDK5 increases NOXA levels through miR-23a repression. J. Biol. Chem..

[31] Jin A., Bao R., Roth M., Liu L., Yang X., Tang X., Yang X., Sun Q., Lu S. (2019). microRNA-23a contributes to asthma by targeting BCL2 in airway epithelial cells and CXCL12 in fibroblasts. Cell. Physiol..

[32] Chen G., Li Y., He Y., Zeng B., Yi C., Wang C., Zhang X., Zhao W., Yu D. (2020). Upregulation of circular RNA circATRNL1 to sensitize oral squamous cell carcinoma to irradiation. Mol. Ther. Nucleic Acids..

[33] Faghihi M.A., Modarresi F., Khalil A.M., Wood D.E., Sahagan B.G., Morgan T.E., Finch C.E., St L.G.R., Kenny P.J., Wahlestedt C. (2008). Expression of a noncoding RNA is elevated in Alzheimer’s disease and drives rapid feed-forward regulation of beta-secretase. Nat. Med..

[34] Keniry A., Oxley D., Monnier P., Kyba M., Dandolo L., Smits G., Reik W. (2012). The H19 lincRNA is a developmental reservoir of miR-675 that suppresses growth and Igf1r. Nat. Cell Biol..

[35] Hennessy E.J., van Solingen C., Scacalossi K.R., Ouimet M., Afonso M.S., Prins J., Koelwyn G.J., Sharma M., Ramkhelawon B., Carpenter S. (2019). The long noncoding RNA CHROME regulates cholesterol homeostasis in primate. Nat. Metab..

[36] Liu T., Zhang H., Zheng J., Lin J., Huang Y., Chen J., Yu Z., Guo L., Pan W., Xiong Y. (2018). SPION-mediated miR-141 promotes the differentiation of HuAESCs into dopaminergic neuron-like cells via suppressing lncRNA-HOTAIR. J. Cell Mol. Med..

[37] Zhao X., Reebye V., Hitchen P., Fan J., Jiang H., Saetrom P., Rossi J., Habib N.A., Huang K.W. (2019). Mechanisms involved in the activation of C/EBPalpha by small activating RNA in hepatocellular carcinoma. Oncogene.

[38] Wang C., Peng R., Zeng M., Zhang Z., Liu S., Jiang D., Lu Y., Zou F. (2020). An autoregulatory feedback loop of miR-21/VMP1 is responsible for the abnormal expression of miR-21 in colorectal cancer cells. Cell Death Dis..

[39] Place R.F., Li L.C., Pookot D., Noonan E.J., Dahiya R. (2008). MicroRNA-373 induces expression of genes with complementary promoter sequences. Proc. Natl. Acad. Sci. USA.

[40] Zhang Y., Liu W., Chen Y., Liu J., Wu K., Su L., Zhang W., Jiang Y., Zhang X., Zhang Y. (2018). A cellular microRNA facilitates regulatory T lymphocyte development by targeting the FOXP3 promoter TATA-box motif. J. Immunol..

[41] Li H., Zhan J., Zhao Y., Fan J., Yuan S., Yin Z., Dai B., Chen C., Wang D.W. (2020). Identification of ncRNA-mediated functions of nucleus-localized miR-320 in cardiomyocytes. Mol. Ther. Nucleic Acids..

[42] Gacita A.M., Fullenkamp D.E., Ohiri J., Pottinger T., Puckelwartz M.J., Nobrega M.A., McNally E.M. (2021). Genetic variation in enhancers modifies cardiomyopathy gene expression and progression. Circulation.

[43] Sucharov C.C., Nakano S.J., Slavov D., Schwisow J.A., Rodriguez E., Nunley K., Medway A., Stafford N., Nelson P., McKinsey T.A. (2019). A PDE3A promoter polymorphism regulates cAMP-induced transcriptional activity in failing human myocardium. J. Am. Coll. Cardiol..

[44] Vahdat-Lasemi M., Hosseini S., Jajarmi V., Kazemi B., Salehi M. (2019). Intraovarian injection of miR-224 as a marker of polycystic ovarian syndrome declines oocyte competency and embryo development. J. Cell. Physiol..

[45] Fortin J., Boehm U., Deng C.X., Treier M., Bernard D.J. (2014). Follicle-stimulating hormone synthesis and fertility depend on SMAD4 and FOXL2. FASEB J..

[46] Guglielmi L., Heliot C., Kumar S., Alexandrov Y., Gori I., Papaleonidopoulou F., Barrington C., East P., Economou A.D., French P. (2021). Smad4 controls signaling robustness and morphogenesis by differentially contributing to the Nodal and BMP pathways. Nat. Commun..

[47] Li X., Du X., Yao W., Pan Z., Li Q. (2020). TGF-beta/SMAD4 signaling pathway activates the HAS2-HA system to regulate granulosa cell state. J. Cell. Physiol..

[48] Monsivais D., Clementi C., Peng J., Titus M.M., Barrish J.P., Creighton C.J., Lydon J.P., DeMayo F.J., Matzuk M.M. (2016). Uterine ALK3 is essential during the window of implantation. Proc. Natl. Acad. Sci. USA.

[49] Du X., Li Q., Cao Q., Wang S., Liu H., Li Q. (2019). Integrated analysis of miRNA-mRNA interaction network in porcine granulosa cells undergoing oxidative stress. Oxid. Med. Cell. Longev..

[50] Zhang J., Gao B., Wang J., Ren Q., Chen J., Ma Q., Zhang Z., Xing B. (2016). Critical role of FoxO1 in granulosa cell apoptosis caused by oxidative stress and protective effects of grape seed procyanidin B2. Oxid. Med. Cell. Longev..

[51] Wang L., Tang J., Wang L., Tan F., Song H., Zhou J., Li F. (2021). Oxidative stress in oocyte aging and female reproduction. J. Cell. Physiol..

[52] Sewastianik T., Szydlowski M., Jablonska E., Bialopiotrowicz E., Kiliszek P., Gorniak P., Polak A., Prochorec-Sobieszek M., Szumera-Cieckiewicz A., Kaminski T.S. (2016). FOXO1 is a TXN- and p300-dependent sensor and effector of oxidative stress in diffuse large B-cell lymphomas characterized by increased oxidative metabolism. Oncogene.

[53] Poulsen R.C., Knowles H.J., Carr A.J., Hulley P.A. (2014). Cell differentiation versus cell death: Extracellular glucose is a key determinant of cell fate following oxidative stress exposure. Cell Death Dis..

[54] Bae J.H., Jeong H.J., Kim H., Leem Y.E., Ryu D., Park S.C., Lee Y.I., Cho S.C., Kang J.S. (2020). ZNF746/PARIS overexpression induces cellular senescence through FoxO1/p21 axis activation in myoblasts. Cell Death Dis..

[55] Zhang C., Tan Z., Xie Y., Zhao Y., Huang T.Y., Lu Z., Luo H., Can D., Xu H., Zhang Y.W. (2019). Appoptosin mediates lesions induced by oxidative stress through the JNK-FoxO1 pathway. Front. Aging Neurosci..

[56] Rajendran N.K., Houreld N.N., Abrahamse H. (2021). Photobiomodulation reduces oxidative stress in diabetic wounded fibroblast cells by inhibiting the FOXO1 signaling pathway. J. Cell Commun. Signal..

[57] Tang Q., Huang K., Liu J., Wu S., Shen D., Dai P., Li C. (2019). Fine particulate matter from pig house induced immune response by activating TLR4/MAPK/NF-kappaB pathway and NLRP3 inflammasome in alveolar macrophages. Chemosphere.

[58] Zhang H., Pan Z., Ju J., Xing C., Li X., Shan M., Sun S. (2020). DRP1 deficiency induces mitochondrial dysfunction and oxidative stress-mediated apoptosis during porcine oocyte maturation. J. Anim. Sci. Biotechnol..

[59] Camerlink I., Bolhuis J.E., Duijvesteijn N., van Arendonk J.A., Bijma P. (2014). Growth performance and carcass traits in pigs selected for indirect genetic effects on growth rate in two environments. J. Anim. Sci..

[60] Chen S.Y., Freitas P., Oliveira H.R., Lazaro S.F., Huang Y.J., Howard J.T., Gu Y., Schinckel A.P., Brito L.F. (2021). Genotype-by-environment interactions for reproduction, body composition, and growth traits in maternal-line pigs based on single-step genomic reaction norms. Genet. Sel. Evol..

[61] Freitas P.H.F., Johnson J.S., Chen S., Oliveira H.R., Tiezzi F., Lázaro S.F., Huang Y., Gu Y., Schinckel A.P., Brito L.F. (2021). Definition of environmental variables and critical periods to evaluate heat tolerance in Large White pigs based on single-step genomic reaction norms. Front. Genet..

[62] Paloviita P., Hyden-Granskog C., Yohannes D.A., Paluoja P., Kere J., Tapanainen J.S., Krjutskov K., Tuuri T., Vosa U., Vuoristo S. (2021). Small RNA expression and miRNA modification dynamics in human oocytes and early embryos. Genome Res..

[63] Kim Y.S., Kim H.R., Kim H., Yang S.C., Park M., Yoon J.A., Lim H.J., Hong S.H., DeMayo F.J., Lydon J.P. (2016). Deficiency in DGCR8-dependent canonical microRNAs causes infertility due to multiple abnormalities during uterine development in mice. Sci. Rep..

[64] Cho S.H., Kim J.H., An H.J., Kim Y.R., Ahn E.H., Lee J.R., Kim J.O., Ko J.J., Kim N.K. (2021). Genetic polymorphisms in miR-604A > G, miR-938G > A, miR-1302-3C > T and the risk of idiopathic recurrent pregnancy loss. Int. J. Mol. Sci..

[65] Lei B., Gao S., Luo L.F., Xia X.Y., Jiang S.W., Deng C.Y., Xiong Y.Z., Li F.E. (2011). A SNP in the miR-27a gene is associated with litter size in pigs. Mol. Biol. Rep..

[66] Przygrodzka E., Sokolowska G., Myszczynski K., Krawczynski K., Kaczmarek M.M. (2020). Clustered microRNAs: The molecular mechanism supporting the maintenance of luteal function during early pregnancy. FASEB J..

[67] Du X., Wang L., Li Q., Wu W., Shang P., Chamba Y., Pan Z., Li Q. (2020). miR-130a/TGF-beta1 axis is involved in sow fertility by controlling granulosa cell apoptosis. Theriogenology.

