# Supplementary material for: A Mutation in Endogenous saRNA miR-23a Influences Granulosa Cells Response to Oxidative Stress
Source: Antioxidants (Basel). 2022 Jun 15;11(6):1174. doi: 10.3390/antiox11061174 (PMC9219974; doi:10.3390/antiox11061174)
Supplement: Supplementary file 1 [file antioxidants-11-01174-s001.zip › antioxidants-1736982-supplementary.pdf]

Supplemental Figures

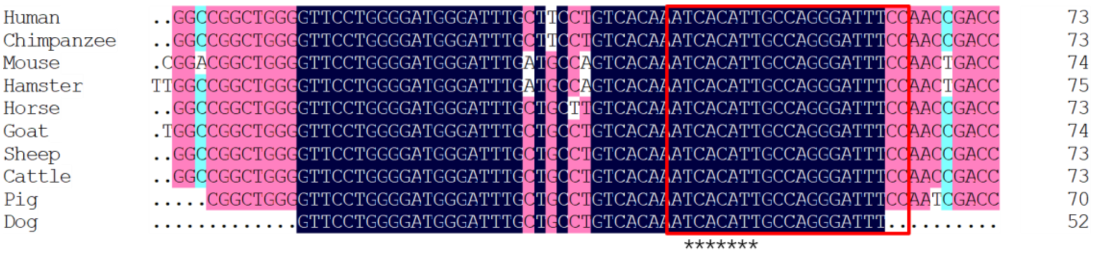

Figure S1. Multi-sequence alignment of pre-miR-23a in mammal. The mature sequence is boxed and the asterisk marks the seed sequence. Black parts represent all species with identical sequences, cyan and magenta represent the same sequence in multiple species.

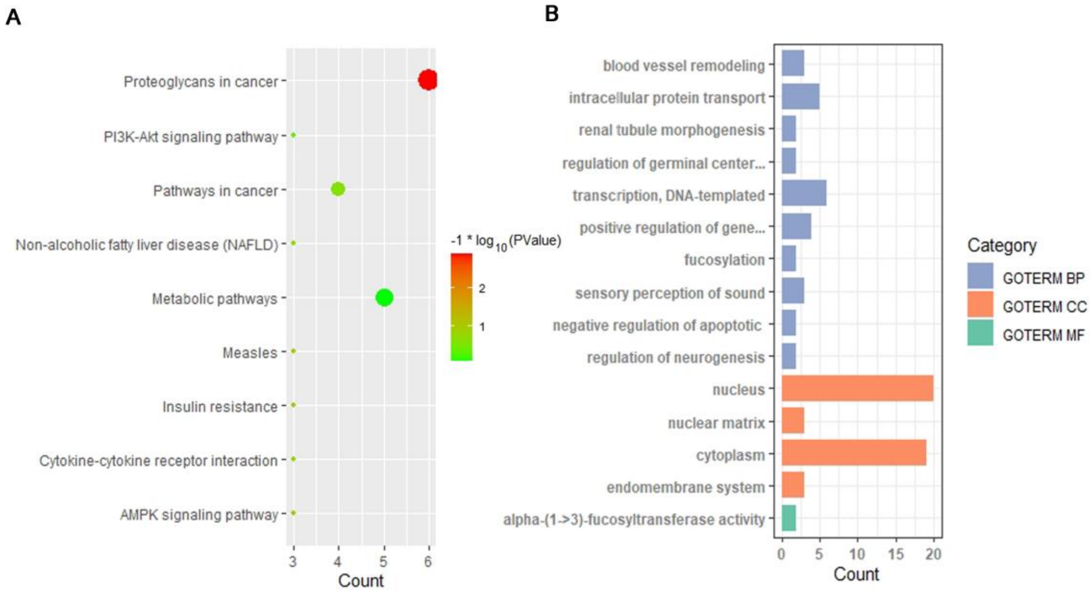

Figure S2. Functional analysis of miR-23a target genes. (**A**) Analysis of KEGG pathway. PValue, Enrichment significance, was calculated by hypergeometric test. -1*Log$_{10}$(PValue) is the ordinate, and the number of enriched genes in each pathway, count, is coordinate. (**B**) GO term analysis. Count is the number of enriched genes in each term. BP means biological process, MF means molecular function, and CC means cellular component.

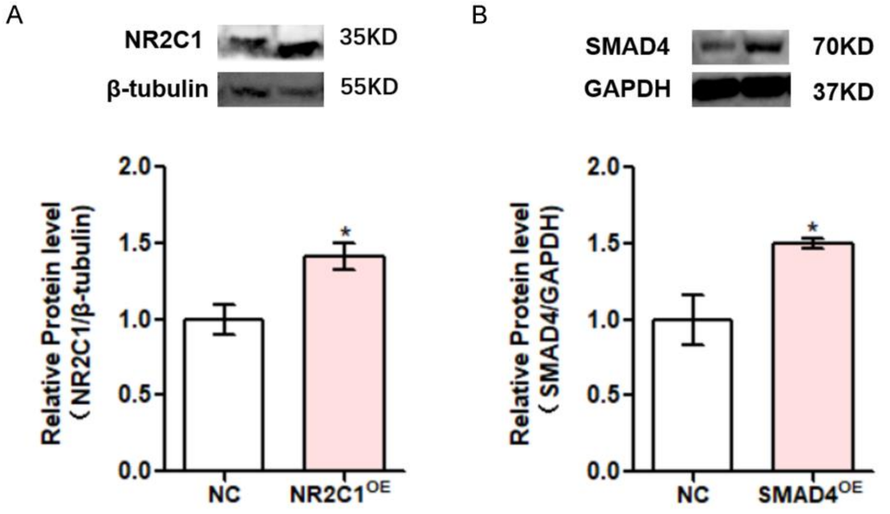

Figure S3. pGCs were treated with pcDNA3.1-SMAD4 and pcDNA3.1-NR2C1, western blotting was performed to detect protein levels of SMAD4 and NR2C1. (**A-B**) β-tubulin and GAPDH are internal reference of NR2C1 (A) and SMAD4 (B), respectively. Data are expressed as mean ± S.E.M., and significance was tested using a t-test. (*P < 0.05).

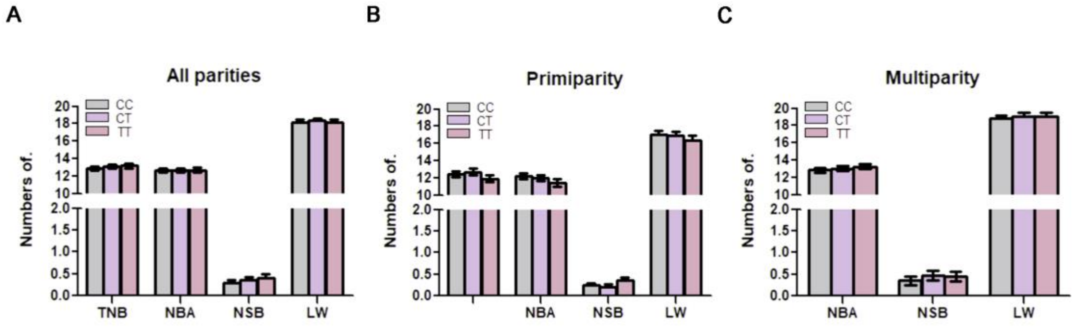

Figure S4. Association analysis of SNV g.-398C>T with reproductive traits in Yorkshire sows. (**A**) All parities. (**B**) Primiparity. (**C**) Multiparity.

Supplemental Tables

Table S1. Information of oligonucleotides.

Oligonucleotides	Accession number	Oligonucleotide sequence (5' to 3')
mimics NC		UUGUACUACACAAAAGUACUG
miR-23a mimics	(NR_038489)	AUCACAUUGCCAGGGAUUUCC
inhibitor NC		CAGUACUUUUGUGUAGUACAA
miR-23a inhibitor	(NR_038489)	GGAAAUCCCUGGCAAUGGAU

Table S2. Information of primers for qRT-PCR.

Name	Accession number	Sequence(5' to 3')	Product size	Annealing temp
miR-23a	NR_038489	F: CGGGCATCACATTGCCAGG<br>R: CAGCCACAAAAGAGCACAAT	56	60
U6	EU520423.1	F: GCTTCGGCAGCACATATACT<br>R: TTCACGAATTTGCGTGTCAT	86	60
NORHA	XR_304632.3	F: GCTGAACCTGGGGAGTGTCTA<br>R: TCTCTGTCCTTGTGGTGTCTGC	176	60
GAPDH	NM_001206359.1	F: GGACTCATGACCACGGTCCAT<br>R: TCAGATCCACAACCGACACGT	163	60
FoxO1	NM_214014.3	F: GCAAATCGAGTTACGGAGGC<br>R: AATGTCATTATGGGGAGGAGAGT	170	60
stem loop	NR_038489	CCTGTTGTCTCCAGCCACAAAAGAGC<br>ACAATATTTCAGGAGACAACAGGGG<br>AAATC		

Table S3. Information of primers for plasmid construction.

Name	Accession number	Primer sequence (5' to 3')	Product size (bp)	Annealing temp (℃)
P189	NC_010444	F: CGGGGTACCCCCTGGCTTTGGGAGTTCCT<br>R: CCGCTCGAGCTGCCATCCTTTGAGGTCTTG	189	56
P558	NC_010444	F: CGGGGTACCGGGAAACTGAGGCTTAAAGGG<br>R: CCGCTCGAGCTGCCATCCTTTGAGGTCTTG	558	58
p1018	NC_010444	F: CGGGGTACCCCCGTCCTAAATCCTTACCAG<br>R: CCGCTCGAGCTGCCATCCTTTGAGGTCTTG	1018	58
p1346	NC_010444	F: CGGGGTACCCCTTCCCGTTCTCGTCCC<br>R: CCGCTCGAGCTGCCATCCTTTGAGGTCTTG	1376	58
pGL3-C/T	NC_010444	F: CGGGGTACCGCCCTGGTGGTTTCCTCCTG<br>R: CCGCTCGAGCTCCCTTCCCCACCACCACT	155	60

Table S4. Information of primer for ChIP.

Name	Accession number	Squence(5' to 3')	Product size (bp)	Annealing temp (℃)
MRE	NC_010449.5	F: TGCTTCGAGGAGCCAGTCTC<br>R: CAGCAGGAGCAGGAAGAAGC	170	55
X	NC_010449.5	F: CCTTCCTAATCCACCGACTT<br>R: GAGGCACGCTCTGAGATTC	144	62
SBE	NC_010444	F: GCCCTGGTGGTTTCCTCCT<br>R: TCCCTTCCCCACCACCACT	154	63

Table S5. Information of primers for Genotyping.

Gene	Accession number	Primer sequences	Product size (bp)	Annealing temp (℃)
miR-23a	NC_010444	F: AGGGCACCGAGTTTTCATT<br>R: ATCAACTGTGTTTCAGCTCAGTAG	1173	58

Table S6. The potential targets of miR-23a.

Targets	Full name
ALAS1	5'-aminolevulinate synthase 1
ANKRD50	ankyrin repeat domain 50
ANXA2	annexin A2
ATP11B	ATPase phospholipid transporting 11B (putative)
ATXN1	ataxin 1
ATXN7L3B	ataxin 7 like 3B
BICD2	BICD cargo adaptor 2
BLCAP	BLCAP, apoptosis inducing factor
BTAF1	B-TFIID TATA-box binding protein associated factor 1
BTBD7	BTB domain containing 7
C2orf69	chromosome 2 open reading frame 69
C6orf62	chromosome 6 open reading frame 62
CADM3	cell adhesion molecule 3
CCDC6	coiled-coil domain containing 6
CDK17	cyclin dependent kinase 17
CELF1	CUGBP Elav-like family member 1
CHD7	chromodomain helicase DNA binding protein 7
CLCN3	chloride voltage-gated channel 3
COL4A1	collagen type IV alpha 1 chain
CREBZF	CREB/ATF bZIP transcription factor
CUL3	cullin 3
CXCL12	C-X-C motif chemokine ligand 12
DCP2	decapping mRNA 2
DHX15	DEAH-box helicase 15
DUSP5	dual specificity phosphatase 5
EGLN2	egl-9 family hypoxia inducible factor 2
EPAS1	endothelial PAS domain protein 1
EPS15	epidermal growth factor receptor pathway substrate 15
ERBB4	erb-b2 receptor tyrosine kinase 4
ETNK1	ethanolamine kinase 1
FAM126B	family with sequence similarity 126 member B
FUT4	fucosyltransferase 4
FUT9	fucosyltransferase 9
HDAC7	histone deacetylase 7
HMGB2	high mobility group box 2
HS6ST3	heparan sulfate 6-O-sulfotransferase 3
IPO5	importin 5
JARID2	jumonji and AT-rich interaction domain containing 2
KIAA1109	KIAA1109
LBR	lamin B receptor
LMNB1	lamin B1
LPAR1	lysophosphatidic acid receptor 1
LRIG1	leucine rich repeats and immunoglobulin like domains 1
MAP3K9	mitogen-activated protein kinase kinase kinase 9
MAPRE1	microtubule associated protein RP/EB family member 1
MARCKS	myristoylated alanine rich protein kinase C substrate
MARCKSL1	MARCKS like 1
MCFD2	multiple coagulation factor deficiency 2
MED13L	mediator complex subunit 13 like
MEF2C	myocyte enhancer factor 2C

MSMO1	methylsterol monooxygenase 1
NAA50	N(alpha)-acetyltransferase 50, NatE catalytic subunit
NAP1L5	nucleosome assembly protein 1 like 5
NCOA6	nuclear receptor coactivator 6
NUAK1	NUAK family kinase 1
ORMDL1	ORMDL sphingolipid biosynthesis regulator 1
OSBPL8	oxysterol binding protein like 8
PARD6B	par-6 family cell polarity regulator beta
PBRM1	polybromo 1
PDE7A	phosphodiesterase 7A
PDPK1	3-phosphoinositide dependent protein kinase 1
PGRMC2	progesterone receptor membrane component 2
PHYHIPL	phytanoyl-CoA 2-hydroxylase interacting protein like
PIK3C2A	phosphatidylinositol-4-phosphate 3-kinase catalytic subunit type 2 alpha
PIK3R3	phosphoinositide-3-kinase regulatory subunit 3
PIP4K2B	phosphatidylinositol-5-phosphate 4-kinase type 2 beta
PKIA	cAMP-dependent protein kinase inhibitor alpha
PKP4	plakophilin 4
PNMA2	PNMA family member 2
PNRC1	proline rich nuclear receptor coactivator 1
POM121C	POM121 transmembrane nucleoporin C
PPP1R12A	protein phosphatase 1 regulatory subunit 12A
PSD3	pleckstrin and Sec7 domain containing 3
PTP4A2	protein tyrosine phosphatase type IVA, member 2
QKI	QKI, KH domain containing RNA binding
QSER1	glutamine and serine rich 1
RAI14	retinoic acid induced 14
RCHY1	ring finger and CHY zinc finger domain containing 1
REPS2	RALBP1 associated Eps domain containing 2
RNF38	ring finger protein 38
RPRD2	regulation of nuclear pre-mRNA domain containing 2
SATB1	SATB homeobox 1
SEMA6D	semaphorin 6D
SETD2	SET domain containing 2, histone lysine methyltransferase
SLC1A1	solute carrier family 1 member 1
SLC25A36	solute carrier family 25 member 36
SMS	spermine synthase
SNX27	sorting nexin family member 27
SPRY2	sprouty RTK signaling antagonist 2
STK35	serine/threonine kinase 35
STRN	striatin
STX12	syntaxin 12
SYT4	synaptotagmin 4
TADA1	transcriptional adaptor 1
TAOK1	TAO kinase 1
TARDBP	TAR DNA binding protein
TBC1D15	TBC1 domain family member 15
TBC1D9	TBC1 domain family member 9
TBR1	T-box, brain 1
TCF24	transcription factor 24
TGFBR2	transforming growth factor beta receptor 2
TGFBR3	transforming growth factor beta receptor 3
TLK1	tousled like kinase 1
TNFAIP3	TNF alpha induced protein 3

TNRC6B	trinucleotide repeat containing 6B
USP46	ubiquitin specific peptidase 46
VCAN	versican
VCPIP1	valosin containing protein interacting protein 1
WNK1	WNK lysine deficient protein kinase 1
ZCCHC2	zinc finger CCHC-type containing 2
ZNF292	zinc finger protein 292
